# Coffee Consumption and Cardiovascular Diseases: A Mendelian Randomization Study

**DOI:** 10.3390/nu13072218

**Published:** 2021-06-28

**Authors:** Shuai Yuan, Paul Carter, Amy M. Mason, Stephen Burgess, Susanna C. Larsson

**Affiliations:** 1 Unit of Cardiovascular and Nutritional Epidemiology, Institute of Environmental Medicine, Karolinska Institutet, SE-171 77 Stockholm, Sweden; shuai.yuan@ki.se; 2 Department of Medicine, University of Cambridge, Cambridge CB2 0QQ, UK; pc602@cam.ac.uk; 3 Department of Public Health and Primary Care, University of Cambridge, Cambridge CB1 8RN, UK; am2609@medschl.cam.ac.uk (A.M.M.); sb452@medschl.cam.ac.uk (S.B.); 4 National Institute for Health Research Cambridge Biomedical Research Centre, University of Cambridge, Cambridge CB2 0QQ, UK; 5 MRC Biostatistics Unit, University of Cambridge, Cambridge CB2 0SR, UK; 6 Unit of Medical Epidemiology, Department of Surgical Sciences, Uppsala University, SE-751 85 Uppsala, Sweden

**Keywords:** cardiovascular disease, coffee, mendelian randomization analysis

## Abstract

Coffee consumption has been linked to a lower risk of cardiovascular disease in observational studies, but whether the associations are causal is not known. We conducted a Mendelian randomization investigation to assess the potential causal role of coffee consumption in cardiovascular disease. Twelve independent genetic variants were used to proxy coffee consumption. Summary-level data for the relations between the 12 genetic variants and cardiovascular diseases were taken from the UK Biobank with up to 35,979 cases and the FinnGen consortium with up to 17,325 cases. Genetic predisposition to higher coffee consumption was not associated with any of the 15 studied cardiovascular outcomes in univariable MR analysis. The odds ratio per 50% increase in genetically predicted coffee consumption ranged from 0.97 (95% confidence interval (CI), 0.63, 1.50) for intracerebral hemorrhage to 1.26 (95% CI, 1.00, 1.58) for deep vein thrombosis in the UK Biobank and from 0.86 (95% CI, 0.50, 1.49) for subarachnoid hemorrhage to 1.34 (95% CI, 0.81, 2.22) for intracerebral hemorrhage in FinnGen. The null findings remained in multivariable Mendelian randomization analyses adjusted for genetically predicted body mass index and smoking initiation, except for a suggestive positive association for intracerebral hemorrhage (odds ratio 1.91; 95% CI, 1.03, 3.54) in FinnGen. This Mendelian randomization study showed limited evidence that coffee consumption affects the risk of developing cardiovascular disease, suggesting that previous observational studies may have been confounded.

## 1. Introduction 

Coffee is comprised of many chemicals which produce both protective and detrimental effects on the cardiovascular system [1,2,3]. A high daily intake of caffeine, present in coffee, acutely increases blood pressure, heart rate and arterial stiffness [2], whereas coffee-contained phenolic compounds, trigonelline, quinides and lignans exert long-term impacts of lowering blood pressure, fatty acid and cholesterol synthesis and augmenting antioxidant activity [1]. Conventional observational studies have found that moderate coffee drinking is linked to a lower risk of overall cardiovascular disease [4,5], coronary artery disease [4,6], stroke [7] and heart failure [8]. However, whether these associations are causal is undetermined due to possible residual confounding factors (e.g., from dietary intake, levels of physical activity, etc.) and other biases inherent in studies with an observational design.

The Mendelian randomization (MR) design [9] can improve causal inference by applying genetic variants as instrumental variables (proxies) for an exposure (e.g., coffee consumption) [10]. Results of recent MR studies found limited data in support of a causal association of coffee consumption with stroke [11,12], atrial fibrillation [13] and heart failure [14] but inconsistent findings for coronary artery disease [12,15,16]. Data are limited for associations of genetically predicted coffee consumption in relation to other cardiovascular diseases. Furthermore, coffee consumption shows mild-to-moderate genetic correlations with body mass index (BMI) and smoking behavior [17], which may exert pleiotropic effects and thus bias coffee–CVD association inference in MR analysis. 

The aim of this MR study was to comprehensively investigate the associations of genetically predicted coffee consumption with 15 cardiovascular outcomes and to further examine these associations using the multivariable MR method [18] to rule out pleiotropy from BMI and smoking. 

## 2. Methods

### 2.1. Genetic Instrument Selection

Fifteen genetic variants (i.e., single-nucleotide polymorphisms (SNPs)) related to coffee consumption at *p* < 5 × 10^−8^ were identified from a genome-wide association meta-analysis including up to 375,833 individuals of European ancestry [19]. The linkage disequilibrium across these SNPs were calculated using the 1000 genomes linkage disequilibrium European panel as the reference population [20]. In this MR study, 12 independent SNPs were applied as genetic instruments for coffee consumption after exclusion of three SNPs (rs117692895, rs12699844 and rs4719497 in chromosome 7) in moderate linkage disequilibrium (*r*^2^ > 0.01) (Supplementary Table S1). Rs2472297 in *CYP1A1/2* and rs4410790 in *AHR* showed strong associations with coffee consumption and explained the majority of phenotypic variance. The median consumption of coffee ranged from 1.1 to 2.5 cups per day across included studies. The effect sizes for the SNP–coffee associations were expressed per 1% of increase in coffee consumption in the GWAS meta-analysis and were rescaled to 50% of increase in the present MR study.

### 2.2. Data Sources of Cardiovascular Diseases

We obtained regression coefficients and corresponding standard errors for the association between the 12 coffee-related SNPs and 15 cardiovascular endpoints from the UK Biobank cohort [21], which consists of approximately 500,000 men and women who were 37 to 73 years of age between 2006–2010. In the present study, we used data from a sample of 367,561 individuals of European ancestry after exclusion of participants with non-European ethnicities, those with high relatedness, excess heterozygosity and low genotype call rate. The cardiovascular endpoints were defined by codes from the 9th and 10th versions of the International Classification of Disease, procedure codes for surgery and self-reported information verified by interview with a nurse (Supplementary Table S2). Cases were ascertained until 30 June 2020. The numbers of cases ranged from 601 for thoracic aortic aneurysm to 35,979 for coronary artery disease. We estimated the genetic associations with cardiovascular disease using logistic regression and the associations were adjusted for age, sex and ten principal components. 

We replicated coffee–cardiovascular disease associations using summary-level data for cardiovascular outcomes from results of GWAS on the FinnGen consortium R4 release [22], including up to 176,899 individuals of Finnish descent. Cardiovascular cases were defined by codes from the 8th and 10th versions of the International Classification of Disease, and surgery and medicine purchase codes from nationwide registries (Supplementary Table S3). Data on abdominal and thoracic aortic aneurysm were not available as separate outcomes but only as aortic aneurysm (both outcomes combined). Likewise, data on aortic valve stenosis were unavailable. To replicate the association for aortic valve stenosis in UK Biobank, the non-rheumatic valve disease outcome was used in FinnGen. The present analyses were approved by the Swedish Ethical Review Authority (2019-02793).

### 2.3. Data Sources for BMI and Smoking Initiation

Summary-level statistics for BMI were extracted from a genome-wide association meta-analysis of UK Biobank and Genetic Investigation of ANthropometric Traits consortium with a total sample of up to 806,834 individuals [23]. Summary-level data for smoking initiation were available from a meta-analysis of 29 GWASs comprising 1,232,091 individuals [24]. Age, sex and major genetic principal components were adjusted for association tests.

### 2.4. Statistical Methods 

The inverse-variance weighted method with random-effects [25] was used as the main analysis. This method provides the most accurate estimate but is sensitivity to pleiotropy [25]. Thus, we employed the weighted median approach [26] and MR-Egger regression [27] as sensitivity analyses. The weighted median method can generate consistent causal estimates assuming >50% of the weight comes from valid SNPs [26]. The MR-Egger regression can detect possible pleiotropic effects and provide estimates after correction for pleiotropy [27]. The I^2^ statistic was calculated to assess the degree of heterogeneity [28] across estimates of instruments in one analysis. We used the *p*-value for intercept in MR-Egger to detect the directional pleiotropic effect [27]. To minimize pleiotropy, we performed a sensitivity analysis using rs2472297 in *CYP1A1/2* and rs4410790 in *AHR* as instrumental variables. Considering positive genetic correlations of coffee consumption with BMI and smoking [19], we obtained causal estimates of associations of coffee consumption with cardiovascular disease using the multivariable inverse-variance weighted model [18] with adjustment for BMI and smoking initiation. Given a small overlap (~23%) between exposure and outcome samples, we calculated the F-statistic [29] to assess the strength of the instruments assuming ~0.48% variance in coffee consumption explained by the 12 SNPs. We used the Bonferroni method to adjust for multiple-testing and associations were considered statistically significant at a *p*-value < 0.003 (0.05/15 outcomes). The statistical analyses were conducted using the packages mrrobust [30] in Stata/SE (version 15.0, StataCorp, College Station, TX, USA) and TwoSampleMR [31] in R Software (version 3.6.0, R Core Team, Vienna, Austria).

## 3. Results

**Figure 1:**
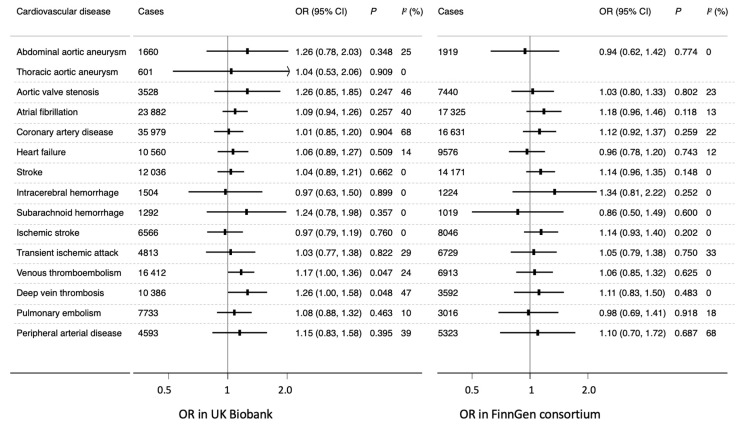
Associations between genetically predicted coffee consumption and cardiovascular diseases. CI indicates confidence interval; OR, odds ratio. The *I*^2^ statistic represents heterogeneity across estimates of used SNPs. Aortic aneurysm includes abdominal and thoracic aortic aneurysm in FinnGen. Data for non-rheumatic valve diseases was used to replicate the association for aortic valve stenosis in FinnGen.

**Figure 2:**
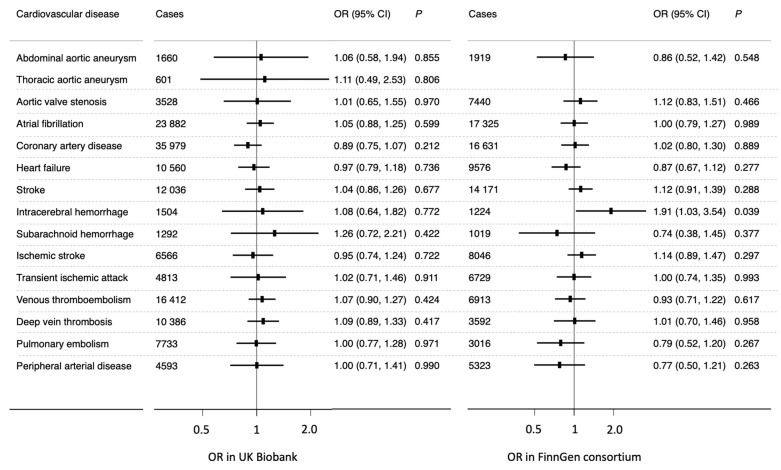
Associations of genetically predicted coffee consumption with cardiovascular diseases in multivariable MR analysis with adjustment for genetically predicted BMI and smoking initiation. CI indicates confidence interval; OR, odds ratio. Aortic aneurysm includes abdominal and thoracic aortic aneurysm in FinnGen. Data for non-rheumatic valve diseases were used in FinnGen to replicate the association for aortic valve stenosis in UK Biobank.

The F-statistic of used SNPs was approximately 148 in the UK Biobank cohort and 71 in FinnGen. Genetically predicted coffee consumption was not associated with any cardiovascular endpoints in either the UK Biobank cohort or in FinnGen in the univariable MR analysis after multiple-testing adjustment (Figure 1). For a 50% increase in genetically predicted coffee consumption, the odds ratio varied from 0.97 (95% confidence interval, 0.63, 1.50) for intracerebral hemorrhage to 1.26 (95% confidence interval, 1.00, 1.58) for deep vein thrombosis in the UK Biobank and from 0.86 (95% confidence interval, 0.50, 1.49) for subarachnoid hemorrhage to 1.34 (95% confidence interval, 0.81, 2.22) for intracerebral hemorrhage in FinnGen. Results were consistent in sensitivity analyses (Supplementary Tables S4 and S5) and the analysis based on rs2472297 and rs4410790 (Supplementary Figure S1). There was mild-to-moderate heterogeneity in certain analyses (Figure 1), whereas no pleiotropy was detected in the MR-Egger regression analysis (*p* for intercept > 0.05) except for the analysis of transient ischemic attack in FinnGen (Supplementary Tables S4 and S5). We detected a suggestive association for intracerebral hemorrhage (odds ratio 1.91; 95% confidence interval, 1.03, 3.54) in FinnGen but no association of genetically predicted coffee consumption with any other cardiovascular diseases in multivariable MR analysis adjusted for BMI and smoking initiation alone (Supplementary Figures S2 and S3) or together (Figure 2).

## 4. Discussion

This univariable MR analysis found limited evidence to support any association of coffee consumption with 15 cardiovascular disease outcomes in the UK Biobank and FinnGen. The multivariable MR analysis, adjusted for BMI and smoking, revealed a suggestive positive association for intracerebral hemorrhage in FinnGen but not for any other cardiovascular diseases in either population.

Our MR findings of no harmful effect of coffee consumption on cardiovascular disease risk corroborate and extend the results of traditional observational studies and previous MR studies of coffee consumption and risk of coronary artery disease [4,6,12,15], heart failure [8,14], atrial fibrillation [13,32,33], ischemic [11] and total stroke [7] as well as a phenome-wide study in the UK Biobank [34]. However, several observational studies have found that moderate coffee consumption (1–5 cups/day), but not heavy coffee consumption, is associated with a reduced risk of coronary artery disease [4,6], heart failure [8], atrial fibrillation [35,36] and total stroke [4,7]. The null findings in our MR study suggest a neutral impact of all bioactive compounds in coffee on cardiovascular disease or a balanced counteraction of cardio-protective and cardio-detrimental components in coffee. Given that this MR study merely examined the linear associations between coffee consumption and cardiovascular diseases, our null findings could not rule out a possible protective effect of moderate but not heavy coffee consumption on certain cardiovascular outcomes, although these inverse associations might be caused by residual confounding, such as protective effects from dietary components and healthy lifestyle factors associated with moderate coffee consumption.

Previous data on coffee consumption and risk of hemorrhagic stroke are scarce and conflicting. As in the present MR study, coffee consumption was not related to risk of subarachnoid hemorrhage in a cohort study of Finnish male smokers [37]. However, a cohort study of Japanese adults found that heavy coffee consumption of over five cups per day was associated with an increased risk of subarachnoid hemorrhage [38], whereas a cohort study of Swedish women found an inverse dose-response association between coffee consumption and risk of subarachnoid hemorrhage (even ≥ five cups per day was related to a reduced risk) [39]. Those Japanese and Swedish cohorts were based on a small number of cases (47 and 79, respectively) and could not rule out chance findings or residual confounding as explanations for the observed associations. Although genetically predicted coffee consumption showed no association with subarachnoid hemorrhage in this MR study, we observed a suggestive positive association for intracerebral hemorrhage in FinnGen but not in UK Biobank after adjustment for BMI and smoking initiation. A previous MR study based on consortium data (not including FinnGen or UK Biobank) also found a suggestive positive association between genetically predicted coffee consumption and intracerebral hemorrhage [11]. Observational studies have found no relation between coffee consumption and risk of intracerebral hemorrhage [37,39]. These previous null findings in observational studies, in combination with inconsistent results for coffee consumption and intracerebral hemorrhage in the present study, suggest that this association may be a chance finding. However, caffeine-containing medications have previously been associated with a 2.2-fold increase risk of intracerebral hemorrhage in a multi-center case-control study in South Korea [40]. Although this study is not directly related, in that it focused on caffeine-containing medications, it adds support for a potential association between caffeinated coffee intake and intracerebral hemorrhage. If present, putative mechanisms could include raised intracranial pressure or hypertension, with coffee known to promote these acutely [41]. Further mechanisms could include endothelial dysfunction or through a raised propensity to bleeding due to inhibition of platelet aggregation by phenolic acid [42]. Further studies are required to clarify the link between coffee consumption and hemorrhagic strokes.

Limited observational data have indicated that coffee consumption might lower the risk of venous thromboembolism [43] and peripheral arterial disease [44] but increase the risk of aortic valve stenosis [45]. The results of the current MR study in two independent European populations did not support any of those associations. A potential explanation for the disparate findings is residual confounding in previous observational studies.

A predominant strength of our MR study is the comprehensive assessment of the relations between genetically predicted coffee consumption and a wide range of cardiovascular diseases in two independent European populations. Moreover, the multivariable MR analysis method [18] was employed to adjust for pleiotropic effects from BMI and smoking, which are genetically correlated with coffee consumption. This restriction of our analyses to European populations minimized bias by population structure, but also limited the generalizability of our findings to other populations.

A limitation of our study is the mild participant overlap in the analyses based on UK Biobank (both the coffee and cardiovascular disease datasets included participants from UK Biobank), which might have resulted in weak instrument bias and model overfitting (leading the MR estimates closer to observational estimates) [29]. Nonetheless, the relatively high F-statistic of the instrument minimized this bias. Certain analyses for outcomes with small numbers of cases might lack statistical power given a small variance explained by the genetic instruments. Thus, we might have overlooked weak associations for the less frequent cardiovascular diseases. Pleiotropy might influence our findings although we did not detect any indication of pleiotropic effects and associations remained consistent in the analysis based on two SNPs, with the strongest association with coffee consumption. Codes to identify aortic stenosis in FinnGen were not available. Although coffee consumption was not associated with non-rheumatic valvular disease, this is a broad range of diseases of differing heart valves, valvular pathologies and etiologies, that may have prevented an association from being found. In addition, coffee consumption patterns might differ between British and Finnish populations. Even though associations were consistent overall in the two populations, this might explain the heterogeneity of associations in the two sources. Another limitation of the present study is that we could not assess whether there is a U-shaped association between coffee consumption and cardiovascular outcomes. Due to heterogeneity in the amount of caffeine and other components in diverse coffee types, the associations with cardiovascular diseases may differ depending on type of coffee consumed. This hypothesis could not be examined in this MR study because genetically predicted coffee consumption is associated with any type of coffee, including both instant and filter coffee as well as Latte, Espresso, other types of coffee and total coffee consumption [46].

Several points need to be investigated in future studies. First, future MR studies are warranted to assess the non-linear association of coffee consumption with cardiovascular disease risk. Second, the associations of individual bioactive components contained in coffee with cardiovascular disease deserves investigation. Third, the impact of different coffee types on cardiovascular disease needs to be examined. Last, whether our findings can be generalized to other populations, such as Asians, needs to be explored.

## 5. Conclusions

The present MR study found no evidence in support of a causal association between coffee consumption and a broad range of cardiovascular outcomes.

## Data Availability

All data analyzed in this study are stored in the OSF data respiratory (https://osf.io/9b27v/, accessed on 16 January 2021).

## Supplementary Materials

The following are available online at https://www.mdpi.com/article/10.3390/nu13072218/s1, Supplementary Table S1. Genetic instruments for coffee consumption; Supplementary Table S2. Diagnostic information on cardiovascular endpoints in UK Biobank; Supplementary Table S3. Diagnostic information on cardiovascular endpoints in FinnGen consortium; Supplementary Table S4. Associations of genetically predicted coffee consumption with cardiovascular diseases in sensitivity analyses in UK Biobank; Supplementary Table S5. Associations of genetically predicted coffee consumption with cardiovascular diseases in sensitivity analyses in FinnGen consortium; Supplementary Figure S1. Associations of genetically predicted coffee consumption with cardiovascular diseases based on rs2472297 and rs4410790; Supplementary Figure S2. Associations of genetically predicted coffee consumption with cardiovascular diseases in multivariable MR analysis with adjustment for body mass index; Supplementary Figure S3. Associations of genetically predicted coffee consumption with cardiovascular diseases in multivariable MR analysis with adjustment for smoking initiation.
