# Supplementary material for: Coffee Consumption and Cardiovascular Diseases: A Mendelian Randomization Study
Source: Nutrients. 2021 Jun 28;13(7):2218. doi: 10.3390/nu13072218 (PMC8308456; doi:10.3390/nu13072218)
Supplement: Supplementary file 1 [file nutrients-13-02218-s001.zip › nutrients-1266556-supplementary.pdf]

Supporting information for

Coffee consumption and cardiovascular disease: a Mendelian randomization study

Shuai Yuan, Amy M. Mason, Paul Carter, Stephen Burgess, Susanna C. Larsson

Supplementary Table S1. Genetic instruments for coffee consumption
Supplementary Table S2. Diagnostic information on cardiovascular endpoints in UK Biobank
Supplementary Table S3. Diagnostic information on cardiovascular endpoints in FinnGen consortium
Supplementary Table S4. Associations of genetically predicted coffee consumption with cardiovascular diseases in sensitivity analyses in UK Biobank
Supplementary Table S5. Associations of genetically predicted coffee consumption with cardiovascular diseases in sensitivity analyses in FinnGen consortium

Supplementary Figure S1. Associations of genetically predicted coffee consumption with cardiovascular diseases based on rs2472297 and rs4410790
Supplementary Figure S2. Associations of genetically predicted coffee consumption with cardiovascular diseases in multivariable MR analysis with adjustment for body mass index
Supplementary Figure S3. Associations of genetically predicted coffee consumption with cardiovascular diseases in multivariable MR analysis with adjustment for smoking initiation

Supplementary Table S1. Genetic instruments for coffee consumption

SNP	pos_hg19	EA	NEA	EAF	Beta	SE	P	Nearby gene
rs597045	11:56272114	A	T	0.69	1.07	0.16	6.62E-11	*OR8U8*
rs1956218	14:33075243	G	A	0.56	0.82	0.15	3.62E-08	*AKAP6*
rs2472297	15:75027880	T	C	0.27	4.54	0.17	5.19E-155	*CYP1A1/2*
rs66723169	18:57808978	A	C	0.23	1.47	0.18	9.88E-17	*MC4R*
rs574367	01:177873210	T	G	0.21	1.05	0.18	8.06E-09	*SEC16B*
rs2330783	22:24747031	G	T	0.99	4.53	0.63	1.57E-12	*SPECC1L-ADORA2A*
rs1260326	02:27730940	C	T	0.61	1.36	0.15	2.62E-19	*GCKR*
rs10865548	02:631606	G	A	0.83	1.54	0.19	4.46E-15	*TMEM18*
rs4410790	07:17284577	C	T	0.63	3.94	0.15	5.59E-141	*AHR*
rs73073176	07:17562952	C	T	0.87	2.31	0.22	5.56E-25	*LOC101927630*
rs34060476	07:73037956	G	A	0.13	1.89	0.22	5.06E-18	*MLXIPL*
rs1057868	07:75615006	T	C	0.29	1.97	0.16	5.26E-33	*POR*

EA, effect allele; EAF, effect allele frequency; NEA, non-effect allele; SE, standard error; SNP, single nucleotide polymorphism.

Supplementary Table S2. Diagnostic information on cardiovascular endpoints in UK Biobank

Outcome	ICD-9 diagnosis	ICD-10 diagnosis	OPCS procedure	Self-report†
Abdominal aneurysm	441.3, 441.4	I71.3, I71.4	L19.4, L19.5	20002
Atrial fibrillation	427.3	I48	-	20002
Aortic valve stenosis	-	I35.0, I35.2	-	20002
Coronary artery disease	410, 411, 412, 414.0, 414.8, 414.9	I21, I22, I23, I24, I25.1, I25.2, I25.5, I25.6, I25.8, I25.9	K40, K41, K42, K43, K44, K45, K46, K49, K50.1, K50.2, K50.4, K75	20002, 20004, 6150
Deep vein thrombosis	451.1	I80.2	L90.2	20002, 6152
Heart failure	402.01, 402.11, 402.91, 404.01, 404.11, 404.91, 404.03, 404.13, 404.93, 428	I11.0, I13.0, I13.2, I50	-	20002
Intracerebral hemorrhage	431	I61	-	20002
Ischemic stroke	434, 436	I63, I64	-	20002
Peripheral arterial disease	443.8, 443.9	I73.8, I73.9	-	20002
Pulmonary embolism	415.1	I26	-	20002, 6152
Stroke	430.X, 431.X, 434.X, 436.X	I60.X, I61.X, I63.X, I64.X	-	1081, 1086, 1491, 1583
Subarachnoid hemorrhage	430	I60	-	20002
Thoracic aortic aneurysm	441.1, 441.2	I71.1, I71.2	-	20002
Transient ischemic attack	435	G45	-	20002
Venous thromboembolism	415.1, 451.1, 452, 453.0, 453.4, 453.9	I26, I80.1, I80.2, I81, I82.0	L90.2	20002, 6152

ICD, International Classification of Disease; OPCS, Office of Population Censuses and Surveys Classification of Surgical Operations and Procedures.
†Numbers refer to data codes used in UK Biobank: 6150/6152 = Health condition diagnosed by doctor (self-reported); 6177 = Medication for health condition (self-reported); 20002 = Non-cancer illness code (self-reported from interview with nurse); 20004 = Surgical operation code (self-reported from interview with nurse).

Supplementary Table S3. Diagnostic information on cardiovascular endpoints in FinnGen consortium

Outcome	ICD-10 diagnosis	ICD-9 diagnosis	ICD-8 diagnosis	KELA reimboursements codes	Medicine purchases: ATC codes
Aortic aneurysm	I718/I713/I711/I712/ I716/I719/I714/I715	0930A	0930|441	-	-
Atrial fibrillation	I48	-	42792	207|351	-
Non-rheumatic valve diseases	I35/I34/I36/I37	424	39[4-6]|424, exclude 4249|3940|3950|3960|3970	-	-
Coronary artery disease	I22/I200/I21/I46/I25/ I23/R96/I21/R98/I24	410/411/412/413/414/798, exclude 7980A	41[0-4]|798	-	-
Deep vein thrombosis	I803	4510A/4511A/4512A	4510	-	-
Heart failure	I50/I130/I132/I110	4029B/428	42700|42710|428|7824	-	C03CA01|C03EB01
Intracerebral hemorrhage	I61	431	431	-	-
Ischemic stroke	I63/I64, exclude I636	4330A/4331A/4339A/ 4340A/4341A/4349A/436	433|434|436	-	-
Peripheral arterial disease	E105/E145/E135/I739/ E125/E115/I702	-	25006|4402|4439	-	-
Pulmonary embolism	I26	415	450	-	-
Subarachnoid hemorrhage	I60	430	430	-	-
Stroke	I60/I61/I62/I63/I64/G45	430/431/4330A/4331A/4339A/ 4340A/4341A/4349A/435/436	430|431|433|434|435|436	-	-
Transient ischemic attack	G45	435	435	-	-
Venous thromboembolism	O882/I80/O871/I26, exclude I800	415/451, exclude 4510	450|451|671|6739	-	-

Diagnostic information was extracted from national registries.

Supplementary Table S4. Associations of genetically predicted coffee consumption with cardiovascular diseases in sensitivity analyses in UK Biobank

Cardiovascular disease	Weighted median method			MR-Egger regression			
	OR	95% CI	*P*	OR	95% CI	*P*	$P_{intercept}$
Abdominal aortic aneurysm	1.23	0.67-2.27	0.507	0.81	0.32-2.01	0.643	0.264
Thoracic aortic aneurysm	0.98	0.42-2.28	0.957	1.42	0.38-5.31	0.600	0.587
Aortic valve stenosis	1.23	0.86-1.77	0.254	1.26	0.58-2.76	0.558	0.987
Atrial fibrillation	1.03	0.89-1.19	0.726	0.98	0.73-1.31	0.881	0.394
Coronary artery disease	0.95	0.81-1.12	0.553	1.00	0.71-1.41	0.987	0.929
Heart failure	1.09	0.88-1.35	0.453	0.92	0.65-1.29	0.616	0.324
Stroke	1.05	0.87-1.27	0.605	1.16	0.86-1.57	0.335	0.388
Intracerebral hemorrhage	0.95	0.53-1.71	0.875	0.85	0.37-1.96	0.699	0.708
Subarachnoid hemorrhage	1.32	0.75-2.33	0.337	1.79	0.73-4.38	0.206	0.357
Ischemic stroke	0.91	0.70-1.19	0.501	0.89	0.60-1.34	0.580	0.645
Transient ischemic attack	0.93	0.69-1.27	0.655	0.96	0.54-1.73	0.902	0.784
Venous thromboembolism	1.07	0.90-1.27	0.454	0.99	0.74-1.32	0.937	0.181
Deep vein thrombosis	1.14	0.93-1.41	0.210	0.95	0.63-1.44	0.809	0.120
Pulmonary embolism	0.96	0.75-1.24	0.752	0.93	0.62-1.38	0.714	0.385
Peripheral arterial disease	1.09	0.79-1.48	0.606	1.07	0.56-2.03	0.843	0.794

CI indicates confidence interval; OR, odds ratio.
$P_{intercept}$ presents the p value for the intercept in MR-Egger method and the $P_{intercept}$ >0.05 indicates no pleiotropy.

Supplementary Table S5. Associations of genetically predicted coffee consumption with cardiovascular diseases in sensitivity analyses in FinnGen consortium

Cardiovascular disease	Weighted median method			MR-Egger regression			
	OR	95% CI	*P*	OR	95% CI	*P*	$P_{intercept}$
Aortic aneurysm	1.02	0.61-1.7	0.945	1.02	0.45-2.32	0.967	0.829
Non-rheumatic valve diseases	1.03	0.78-1.37	0.834	1.32	0.81-2.14	0.266	0.252
Atrial fibrillation	1.11	0.86-1.43	0.419	0.93	0.63-1.38	0.731	0.165
Coronary artery disease	1.13	0.91-1.4	0.265	1.07	0.72-1.6	0.729	0.804
Heart failure	0.92	0.71-1.2	0.559	0.89	0.57-1.37	0.587	0.658
Stroke	1.20	0.97-1.48	0.098	1.44	1.03-2	0.034	0.109
Intracerebral hemorrhage	1.66	0.88-3.14	0.118	2.29	0.86-6.05	0.096	0.210
Subarachnoid hemorrhage	0.68	0.33-1.39	0.286	0.48	0.17-1.39	0.176	0.206
Ischemic stroke	1.21	0.94-1.56	0.144	1.39	0.93-2.06	0.105	0.262
Transient ischemic attack	1.15	0.87-1.52	0.324	1.74	1.13-2.69	0.012	0.007
Venous thromboembolism	1.00	0.76-1.31	0.985	1.01	0.66-1.55	0.957	0.816
Deep vein thrombosis	1.19	0.81-1.76	0.371	0.91	0.51-1.62	0.740	0.417
Pulmonary embolism	0.84	0.56-1.26	0.396	0.96	0.47-2	0.923	0.957
Peripheral arterial disease	0.90	0.61-1.31	0.571	0.98	0.4-2.43	0.966	0.777

CI indicates confidence interval; OR, odds ratio.
$P_{intercept}$ presents the p value for the intercept in MR-Egger method and the $P_{intercept}$ >0.05 indicates no pleiotropy.
Aortic aneurysm included abdominal and thoracic aortic aneurysm in FinnGen. Data for non-rheumatic valve diseases was used to replicate the association for aortic valve stenosis in FinnGen.

Supplementary Figure S1. Associations of genetically predicted coffee consumption with cardiovascular diseases based on rs2472297 and rs4410790

Source	CVD	OR (95% CI)	P-value
UKBB	Abdominal aortic aneurysm	1.25 (0.67, 2.33)	0.482
UKBB	Atrial fibrillation	1.01 (0.85, 1.20)	0.916
UKBB	Aortic valve stenosis	1.29 (0.84, 1.98)	0.241
UKBB	Coronary artery disease	1.02 (0.88, 1.18)	0.779
UKBB	Deep vein thrombosis	1.17 (0.91, 1.51)	0.208
UKBB	Heart failure	0.98 (0.76, 1.26)	0.895
UKBB	Intracerebral hemorrhage	0.92 (0.48, 1.76)	0.804
UKBB	Ischemic stroke	0.88 (0.64, 1.20)	0.410
UKBB	Peripheral arterial disease	1.21 (0.83, 1.75)	0.328
UKBB	Pulmonary embolism	0.91 (0.68, 1.21)	0.512
UKBB	Subarachnoid hemorrhage	1.43 (0.71, 2.89)	0.312
UKBB	Stroke	1.06 (0.84, 1.34)	0.641
UKBB	Thoracic aortic aneurysm	1.26 (0.45, 3.52)	0.659
UKBB	Transient ischemic attack	0.96 (0.66, 1.38)	0.812
UKBB	Venous thromboembolism	1.06 (0.87, 1.30)	0.553
FinnGen	Aortic aneurysm	1.02 (0.55, 1.89)	0.941
FinnGen	Atrial fibrillation	1.09 (0.81, 1.46)	0.572
FinnGen	Aortic valve stenosis	1.02 (0.73, 1.42)	0.914
FinnGen	Coronary artery disease	1.15 (0.89, 1.50)	0.286
FinnGen	Deep vein thrombosis	1.02 (0.65, 1.60)	0.927
FinnGen	Heart failure	0.90 (0.67, 1.22)	0.506
FinnGen	Intracerebral hemorrhage	2.00 (0.94, 4.25)	0.073
FinnGen	Ischemic stroke	1.24 (0.91, 1.69)	0.164
FinnGen	Peripheral arterial disease	1.04 (0.71, 1.53)	0.832
FinnGen	Pulmonary embolism	0.80 (0.49, 1.31)	0.380
FinnGen	Subarachnoid hemorrhage	0.63 (0.28, 1.43)	0.270
FinnGen	Stroke	1.27 (0.98, 1.64)	0.072
FinnGen	Transient ischemic attack	1.22 (0.87, 1.70)	0.254
FinnGen	Venous thromboembolism	0.98 (0.71, 1.36)	0.910

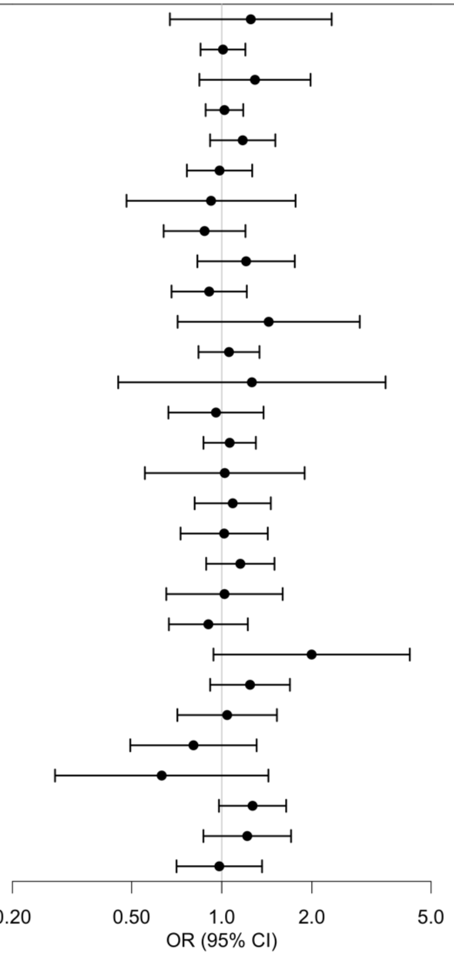

CI, confidence interval; CVD, cardiovascular disease; OR, odds ratio. Aortic aneurysm included abdominal and thoracic aortic aneurysm in FinnGen**.**

Supplementary Figure S2. Associations of genetically predicted coffee consumption with cardiovascular diseases in multivariable Mendelian randomization analysis with adjustment for body mass index

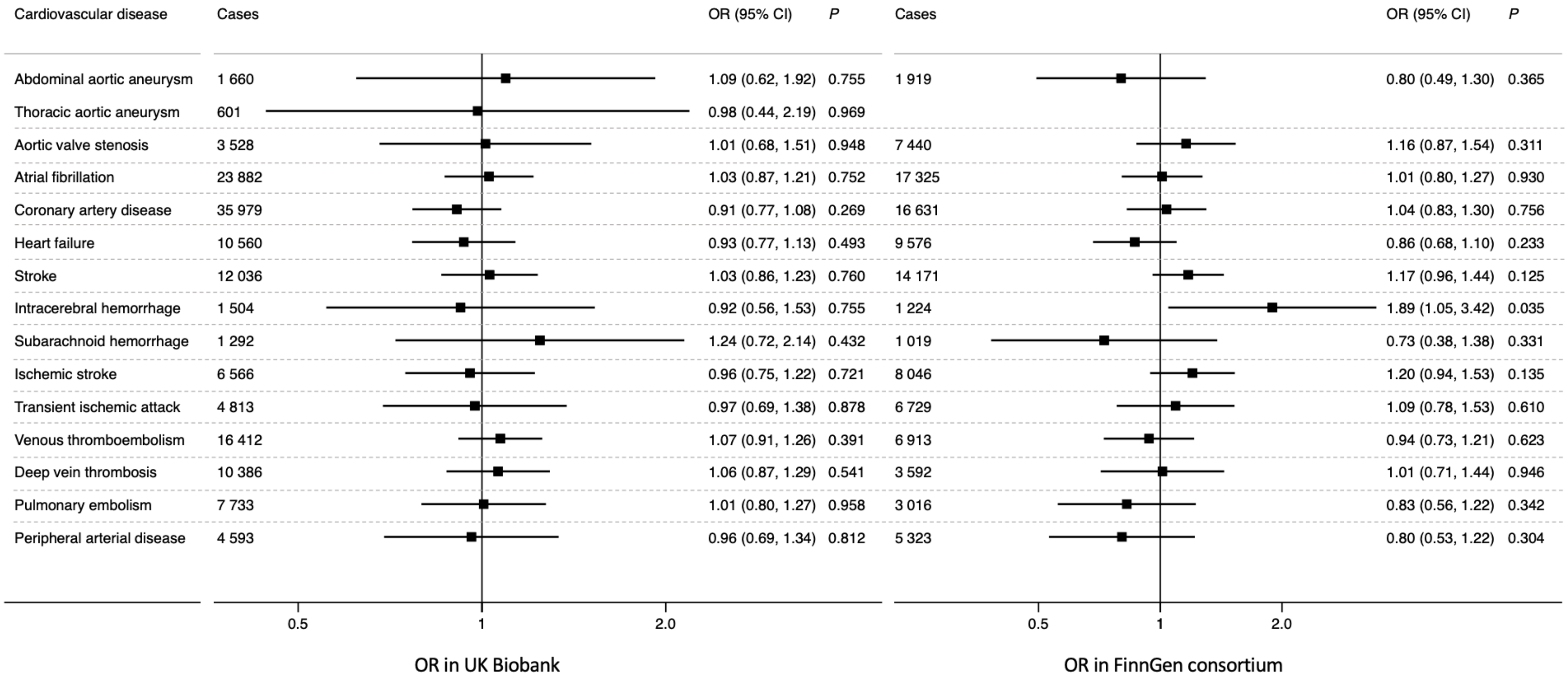

CI indicates confidence interval; OR, odds ratio.
Aortic aneurysm included abdominal and thoracic aortic aneurysm in FinnGen. Data for non-rheumatic valve diseases was used to replicate the association for aortic valve stenosis in FinnGen.

Supplementary Figure S3. Associations of genetically predicted coffee consumption with cardiovascular diseases in multivariable Mendelian randomization analysis with adjustment for smoking initiation

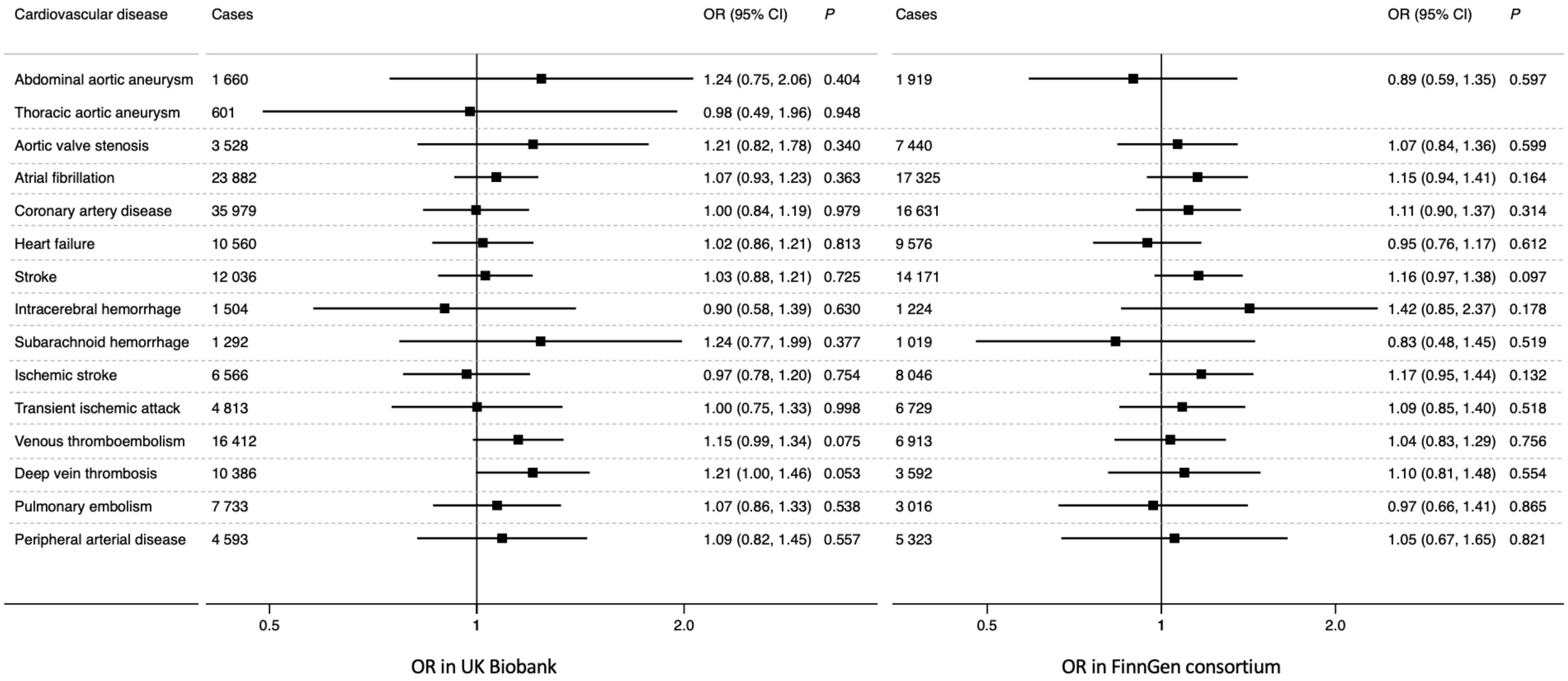

Cardiovascular disease	Cases (UK Biobank)	OR (95% CI)	*P*	Cases (FinnGen)	OR (95% CI)	*P*
Abdominal aortic aneurysm	1 660	1.24 (0.75, 2.06)	0.404	1 919	0.89 (0.59, 1.35)	0.597
Thoracic aortic aneurysm	601	0.98 (0.49, 1.96)	0.948			
Aortic valve stenosis	3 528	1.21 (0.82, 1.78)	0.340	7 440	1.07 (0.84, 1.36)	0.599
Atrial fibrillation	23 882	1.07 (0.93, 1.23)	0.363	17 325	1.15 (0.94, 1.41)	0.164
Coronary artery disease	35 979	1.00 (0.84, 1.19)	0.979	16 631	1.11 (0.90, 1.37)	0.314
Heart failure	10 560	1.02 (0.86, 1.21)	0.813	9 576	0.95 (0.76, 1.17)	0.612
Stroke	12 036	1.03 (0.88, 1.21)	0.725	14 171	1.16 (0.97, 1.38)	0.097
Intracerebral hemorrhage	1 504	0.90 (0.58, 1.39)	0.630	1 224	1.42 (0.85, 2.37)	0.178
Subarachnoid hemorrhage	1 292	1.24 (0.77, 1.99)	0.377	1 019	0.83 (0.48, 1.45)	0.519
Ischemic stroke	6 566	0.97 (0.78, 1.20)	0.754	8 046	1.17 (0.95, 1.44)	0.132
Transient ischemic attack	4 813	1.00 (0.75, 1.33)	0.998	6 729	1.09 (0.85, 1.40)	0.518
Venous thromboembolism	16 412	1.15 (0.99, 1.34)	0.075	6 913	1.04 (0.83, 1.29)	0.756
Deep vein thrombosis	10 386	1.21 (1.00, 1.46)	0.053	3 592	1.10 (0.81, 1.48)	0.554
Pulmonary embolism	7 733	1.07 (0.86, 1.33)	0.538	3 016	0.97 (0.66, 1.41)	0.865
Peripheral arterial disease	4 593	1.09 (0.82, 1.45)	0.557	5 323	1.05 (0.67, 1.65)	0.821

CI indicates confidence interval; OR, odds ratio.
Aortic aneurysm included abdominal and thoracic aortic aneurysm in FinnGen. Data for non-rheumatic valve diseases was used to replicate the association for aortic valve stenosis in FinnGen.
